# Impact of integrative energy healing on anxiety among individuals with cancer

**DOI:** 10.6026/973206300211755

**Published:** 2025-06-30

**Authors:** Gurav Priyankaben Rajeshbhai, Mahalakshmi B., Siva Subramanian N.

**Affiliations:** 1 Department of Pediatric Nursing, Nootan College of Nursing, Sankalchand Patel University, Visnagar, Gujarat - 384315; India; 2 Department of Psychiatric Nursing, Nootan College of Nursing, Sankalchand Patel University, Visnagar ,Gujarat - 384315; India

**Keywords:** Integrative Energy Healing therapy, anxiety, adolescents, cancer, complementary therapy

## Abstract

Cancer diagnosis during adolescence can be overwhelming, often leading to heightened anxiety levels due to physical discomfort, treatment stress and psychological burden. Reiki therapy, an energy-based healing technique, has gained attention as a complementary approach to alleviate anxiety. Therefore, it is of interest to assess the impact of Reiki therapy on anxiety levels among adolescents (aged 13-17) with cancer in selected hospitals in Gujarat. A quasi-experimental pre-test and post-test control group design was used with 92 participants (46 in the intervention group and 46 in the control group). The intervention group received six daily sessions of Reiki therapy (21 minutes each), while the control group received standard care. Anxiety was assessed using the Hamilton Anxiety Rating Scale. Results showed a significant reduction in anxiety scores post-intervention in the Reiki group (Mean difference = 8.58, p < 0.001), whereas the control group did not show statistically significant improvement (p = 0.055). Thus, the effectiveness of Reiki therapy in reducing anxiety among adolescents with cancer is reported advocate its integration into holistic pediatric oncology care.

## Background:

Adolescence is a critical developmental phase where individuals experience not only physical and emotional changes but also build their identity and autonomy. A diagnosis of cancer during this period disrupts this trajectory, often triggering intense emotional disturbances, including anxiety, fear, and uncertainty. Anxiety is a frequent psychological response to life-threatening illnesses such as cancer and is marked by excessive worry, agitation and a range of somatic symptoms including sleep disturbances and cardiovascular changes. Adolescents undergoing cancer treatment are particularly at risk, as the stress of medical procedures, uncertain prognosis and disruption of social and developmental milestones converge to heighten psychological distress [1, 2]. Traditional pharmacologic treatments for anxiety, such as benzodiazepines and SSRIs, though effective, are often accompanied by side effects, dependency risks and limitations for long-term use in adolescents. This has led to increasing interest in non-pharmacologic approaches, particularly complementary and alternative medicine (CAM), to alleviate psychological symptoms in a safer and more sustainable manner [3]. One such CAM intervention is Reiki therapy, a form of energy healing that involves the practitioner channeling energy through gentle touch or near-body hand positions to promote balance in the recipient's biofield. Reiki has gained increasing popularity for its purported benefits in enhancing well-being, relieving stress and reducing symptoms of pain and anxiety [4]. In a randomized trial, Reiki therapy was shown to significantly reduce anxiety scores in cancer patients, with improvements noted even after a single session [5]. Systematic reviews have also highlighted its potential role in symptom management, particularly within oncology and palliative care contexts [6]. For example, Sevcan (2025) reviewed seven randomized trials and found moderate-to-large effect sizes for Reiki's impact on anxiety, particularly among cancer patients [7]. Another recent study by Akpinar *et al.* (2023) reported that Reiki produced significant improvements in both general and physiological manifestations of anxiety, reinforcing its value across diverse healthcare settings [8]. Although the bulk of Reiki research focuses on adults, some preliminary investigations suggest that it may also be effective in younger populations [9]. Therefore, it is of interest to evaluate the impact of Reiki therapy on anxiety among adolescents aged 13-17 years diagnosed with cancer, receiving treatment at selected hospitals in Gujarat.

## Methodology:

## Research design:

This study adopted a quasi-experimental pre-test and post-test control group design to evaluate the effectiveness of Reiki therapy in reducing anxiety among adolescents diagnosed with cancer.

## Research setting:

The study was conducted in selected cancer hospitals across Gujarat, India.

## Sample size:

A total of 92 adolescents, aged between 13 and 17 years, who had been diagnosed with various forms of cancer and were undergoing treatment, were recruited. The participants were divided into two groups: 46 in the intervention group and 46 in the control group.

## Sampling technique:

A convenience sampling method was used to select participants who met the inclusion criteria: adolescents who (1) were clinically diagnosed with cancer, (2) were receiving active treatment, (3) exhibited anxiety symptoms and (4) provided assent along with parental/guardian consent. Patients with critical cognitive impairments or those unwilling to participate were excluded.

## Assessment instrument:

Anxiety levels were measured using the Hamilton Anxiety Rating Scale (HAM-A), a validated tool widely used in both clinical and research settings to assess psychological and somatic symptoms of anxiety. The tool includes 14 items scored on a scale of 0 (not present) to 4 (very severe), with total scores ranging from 0 to 56.

## Therapeutic intervention:

Participants in the intervention group received Reiki therapy from a certified Reiki practitioner. The therapy consisted of six consecutive daily sessions, each lasting 21 minutes, focusing on the body's seven major chakras. The control group received routine medical care without any additional complementary therapy.

## Data analysis strategy:

All collected data were coded and entered into SPSS for statistical analysis. Descriptive statistics were employed to depict demographic variables and baseline clinical characteristics. Inferential statistics included:

[1] Paired t-tests to assess intra-group differences in anxiety scores (pre vs. post),

[2] Independent t-tests to compare mean scores between the control and intervention groups,

[3] Chi-square tests to examine associations between baseline anxiety and selected demographic parameters.

Statistical significance was determined at a p-value < 0.05.

## Results:

Table 1 (see PDF) summarizes the demographic characteristics of the participants. Most children in both groups were aged 13-17 years, with a higher proportion of males. The majority in the experimental group had primary education, belonged to joint families and had a monthly income above ₹20,000, while the control group showed more variation in parental occupation and education levels. Table 2 (see PDF) show Post-test results showed a marked reduction in anxiety in the study group compared to the control group. In the study group, participants with no anxiety increased from 10.8% to 50.0%, while severe anxiety dropped from 28.2% to 8.7%. In contrast, the control group showed minimal improvement, with severe anxiety rising slightly from 30.4% to 32.6% and only 15.2% reporting no anxiety. This indicates that Reiki therapy significantly reduced anxiety levels. Table 3 (see PDF) shows the comparison of mean anxiety scores between the study and control groups before and after the intervention. The study group demonstrated a significant reduction in anxiety scores (Mean Difference = 8.58, p < 0.001), indicating the effectiveness of Reiki therapy, while the control group showed no statistically significant change (p = 0.055). Figure 1 (see PDF) shows the comparison of anxiety levels (No, Minimal, Moderate, Severe) between the experimental and control groups before and after the intervention. The experimental group demonstrated a clear reduction in moderate and severe anxiety and a significant increase in the "No Anxiety" category post-intervention, highlighting the effectiveness of Reiki therapy in reducing anxiety among adolescents with cancer.

## Discussion:

The results of the present study underscore the potential of Integrative Energy Healing therapy as a viable non-pharmacological strategy for managing anxiety in adolescents undergoing cancer treatment. Adolescence is a period marked by psychological upheaval, and a cancer diagnosis during this time can significantly intensify emotional distress. The significant reduction in anxiety levels observed among participants receiving Reiki therapy affirms its therapeutic relevance in supportive oncology care. These findings are consistent with a study by Birocco *et al.*, which reported a substantial decline in anxiety scores in cancer patients following Reiki sessions [10]. Similarly, Dyer *et al.* documented a mean anxiety reduction of 2.09 points after short Reiki interventions in cancer infusion centers [11]. Furthermore, Pattanshetty *et al.* observed decreased pain and anxiety and improved quality of life in cancer survivors receiving daily Reiki sessions [12]. Integrative Energy Healing therapy also shown promise in pediatric oncology and palliative care settings. Thrane *et al.* found medium to large effect sizes for pain and anxiety reduction in children receiving Reiki, supporting its utility in younger populations [13]. Additionally, Lopes-Júnior *et al.* conducted a systematic review and concluded that Reiki was effective in managing symptom clusters like anxiety, worry and dyspnea in pediatric palliative care [14]. Qualitative insights from Gomez and Gonzom reinforced these findings, with patients reporting emotional regulation and calmness post-Reiki [15]. While some researchers express caution, such as Joyce and Herbison in a Cochrane review that called for more high-quality trials to confirm Reiki's effects on anxiety and depression [16], others such as Zadro and Stapleton demonstrated that Reiki had statistically significant effects on mental health beyond placebo in their 2022 review [17]. The efficacy observed in our study also resonates with Guo *et al.*'s meta-analysis, where Reiki was shown to produce sustained relief from anxiety in cancer patients, many of whom expressed willingness to recommend the therapy [18]. This suggests Reiki could be a valuable, scalable intervention in resource-limited oncology care. DiScipio's study further showed that combining Reiki with restorative yoga enhanced relaxation and decreased intrusive thoughts more than yoga alone [19]. In a crossover trial by Tsang *et al.* Reiki significantly reduced fatigue and anxiety among cancer patients, outperforming rest periods [20].

Practical integration of Integrative Energy Healing therapy in healthcare systems has been successfully demonstrated. Rosenbaum and Velde found that among various complementary therapies, Reiki had the highest impact on pain relief and mood improvement in cancer patients [21]. Dyer *et al.* echoed this with strong patient satisfaction in their Reiki volunteer program during chemotherapy infusions [11]. Chirico *et al.* suggested that patients with higher self-efficacy derive greater benefit from Reiki, highlighting the role of psychological readiness in maximizing treatment outcomes [22]. This may be particularly relevant in adolescent populations, where engagement and belief in therapy are critical. Our Reiki protocol emphasized chakra alignment over six sessions, with measurable improvement noted. This is comparable to Souza *et al.*'s findings where even a single Reiki session led to symptom relief in advanced cancer patients [23]. Home-based Reiki, as examined by Chen *et al.*, showed that family caregivers can be trained to deliver effective Reiki, demonstrating feasibility in home settings and offering a scalable model for India's healthcare context [24]. Overall, the study contributes strong evidence for integrating Reiki into adolescent cancer care. Despite some limitations-such as the use of convenience sampling and the inability to blind participants-our structured design and use of validated tools enhance the reliability of the findings. Future multicentric trials with sham controls would further strengthen the case for Reiki as a supportive intervention. In conclusion, Reiki therapy significantly reduces anxiety in adolescents with cancer and aligns well with international literature advocating for non-pharmacological adjuncts in oncology care. Its low cost, safety and positive reception make it a viable tool for enhancing emotional well-being in young cancer patients.
